# Diagnostic Accuracy of Digital Mammography in the Detection of Breast Cancer

**DOI:** 10.7759/cureus.2448

**Published:** 2018-04-08

**Authors:** Muhammad Zeeshan, Basit Salam, Qazi Saad B Khalid, Shahbaz Alam, Raza Sayani

**Affiliations:** 1 Radiology, Royal Blackburn Hospital, Elht; 2 Department of Radiology, The Aga Khan University, Karachi, PAK; 3 Radiology, Dallah Hospital

**Keywords:** breast cancer, digital mammography, spiculated density, pleomorphic microcalcifications, architectural distortion, skin thickening, nipple retraction

## Abstract

Introduction

Breast cancer has a high prevalence in the community and places very high demands on resources. Digital mammography provides a good quality image with reduced radiation dose and can detect breast carcinoma in its earlier stages, resulting in good prognosis and improved patient survival.

Objective

To calculate the diagnostic accuracy of digital mammography in the detection of breast cancer, using histopathology as a gold standard in women aged over 30 years, who are undergoing mammography for screening and diagnostic purposes.

Materials and methods

This was a cross-sectional analytical study, conducted in the department of radiology, for a total duration of 10 months. A total of 122 patients of age above 30 years, referred for digital mammography for the evaluation of different symptoms related to breast diseases, followed by biopsy/surgery and histopathology, were included in the study.

Result

Our data confirmed that digital mammography is a highly accurate tool for breast cancer detection having a sensitivity of 97%, a specificity of 64.5%, a positive predictive value of 89%, and a negative predictive value of 90.9%, with a diagnostic accuracy of 89.3%.

Conclusion

Considering our results, we recommend that digital mammography should replace screen-film mammography as a basic tool to detect breast cancer for both screening and diagnostic purposes.

## Introduction

Breast cancer is the most common cancer in females in the United States and throughout the world. It is responsible for 21% of new cancer cases worldwide [1]. It is also prevalent among women in Pakistan (accounting for one-third of the cancers in females) [2]. The five-year survival of stage IV breast cancer is 10%. However, earlier detection and treatment can improve five-year survival to 85% [1].

A study conducted by Karin Flobbe et al. in the Netherlands has estimated the prevalence of breast cancer to be 6.3%, which is consistent with the results of other studies [3]. Breast cancer detection via mammography has been recommended traditionally and the majority of women older than 40 years in the United States participate [4]. Recent research conducted on 210,000 women in Sweden has shown that in women aged 40-49 years, there was a significant 48% reduction in breast cancer mortality in those exposed to screening, whereas there was none in those unscreened [5].

Both screen-film mammography and digital mammography use x-rays to obtain images. With screen-film mammography, the image is captured on film; with digital mammography, the image is captured digitally [6]. Digital mammography is developed to address the limitations of film mammography and separates image acquisition and display, allowing the optimization of both [7].

In many recent studies, digital mammography was significantly better than conventional film mammography at detecting breast cancer in young women, premenopausal and perimenopausal women, and women with dense breasts [8]. The visibility of a subtle mass or cluster of microcalcifications present in the image can be increased if image contrast is adjusted [9]. Approximately 25-43% of non-palpable cancers are detected on mammography because of microcalcifications [10].

Digital mammography also offers other benefits over film mammography in the form of easier access to images and computer-assisted diagnosis; improved means of transmission, retrieval, and storage of images; and the use of a lower average dose of radiation without compromising diagnostic accuracy [7].

Pisano et al., in one of their studies, calculated the sensitivity and specificity of digital mammography to be 85% and 90%, respectively, in females aged less than 50 years and pre- or perimenopausal women with non-dense breasts [11].

The aim of this study is to calculate the diagnostic accuracy of digital mammography in detecting breast cancer early, using histopathology as the gold standard in women aged over 30 years who are undergoing mammography for screening and diagnostic purposes. As the early detection of breast cancer increases the chances of cure and subsequently improves quality of life, this study will provide a landmark for other institutions to acquire and use digital mammography equipment for screening purposes.

## Materials and methods

This was a cross-sectional analytical study conducted in the department of radiology, for a total duration of 10 months. The sampling technique was non-probability, purposive. The sample size was calculated using sample size determination in the health studies manual by WHO. The reported sensitivity of a digital mammogram to detect breast cancer is 85% and the reported specificity is 90% [11]. The prevalence of breast cancer is 20% (one in five female patients). The desired precision is 12%, so 121 patients were the required sample. We included 122 patients in the study.

All female patients above 30 years of age referred to the radiology department for digital mammography followed by biopsy were included. Patients who were already diagnosed with breast cancer and underwent digital mammography for follow-up and those patients who had inconclusive mammograms due to increased breast density and needed an ultrasound for further evaluation were excluded. Similarly, patients whose histopathology results were not available or they did not undergo biopsy/surgery were also excluded.

Informed consent was taken for the examination and inclusion in the study. After explaining the procedure, a brief history was taken regarding the patient’s signs and symptoms, their duration, and any family history of breast cancer. Mammograms were performed on Mammomat Nova 3000 (Siemens AG, Munich, Germany) at 26-30 Kvp and mAs were set on automatic exposure control. Automatic exposure control is a default setting in digital mammographic equipment to control the exposure and is calibrated by an experienced physicist having a minimum of five years' experience.

Images were acquired in the mediolateral oblique and craniocaudal projections. When needed, additional views, such as exaggerated view, axillary view, magnified view, and cone compression view, were taken. The digital mammograms were analyzed by a post-graduate consultant radiologist with minimum five years' experience, while the histopathology results were provided by a post-graduate consultant pathologist with minimum five years' experience, which served as the gold standard. The findings of digital mammography, histopathology, and demographics were entered in the proforma by the researcher.

The patient’s data were collected using a standard proforma. Data entry and analysis were done through IBM SPSS for Windows, version 19.0 (IBM Corp, Armonk, NY). Descriptive statistics were calculated. Mean and standard deviation were calculated for the age of the patient. Frequencies and percentages were calculated for the site of the lesion in the breast, any family history of breast cancer, the associated pleomorphic microcalcifications, architectural distortion, skin thickening and nipple retraction, and presence or absence of axillary lymph nodes.

Confounding variables, like age, family history of breast cancer, and use of hormone replacement therapy (HRT), were dealt with in data analysis and stratification was done to see the effect of these on the outcome variable.

A 2x2 table was used to calculate sensitivity, specificity, positive predictive value, negative predictive value, and diagnostic accuracy. Sensitivity, specificity, positive and negative predictive values, and diagnostic accuracy of digital mammography in the detection of breast cancer were calculated against histopathology as the gold standard, as defined in the operational definition.

## Results

**Figure 1:**
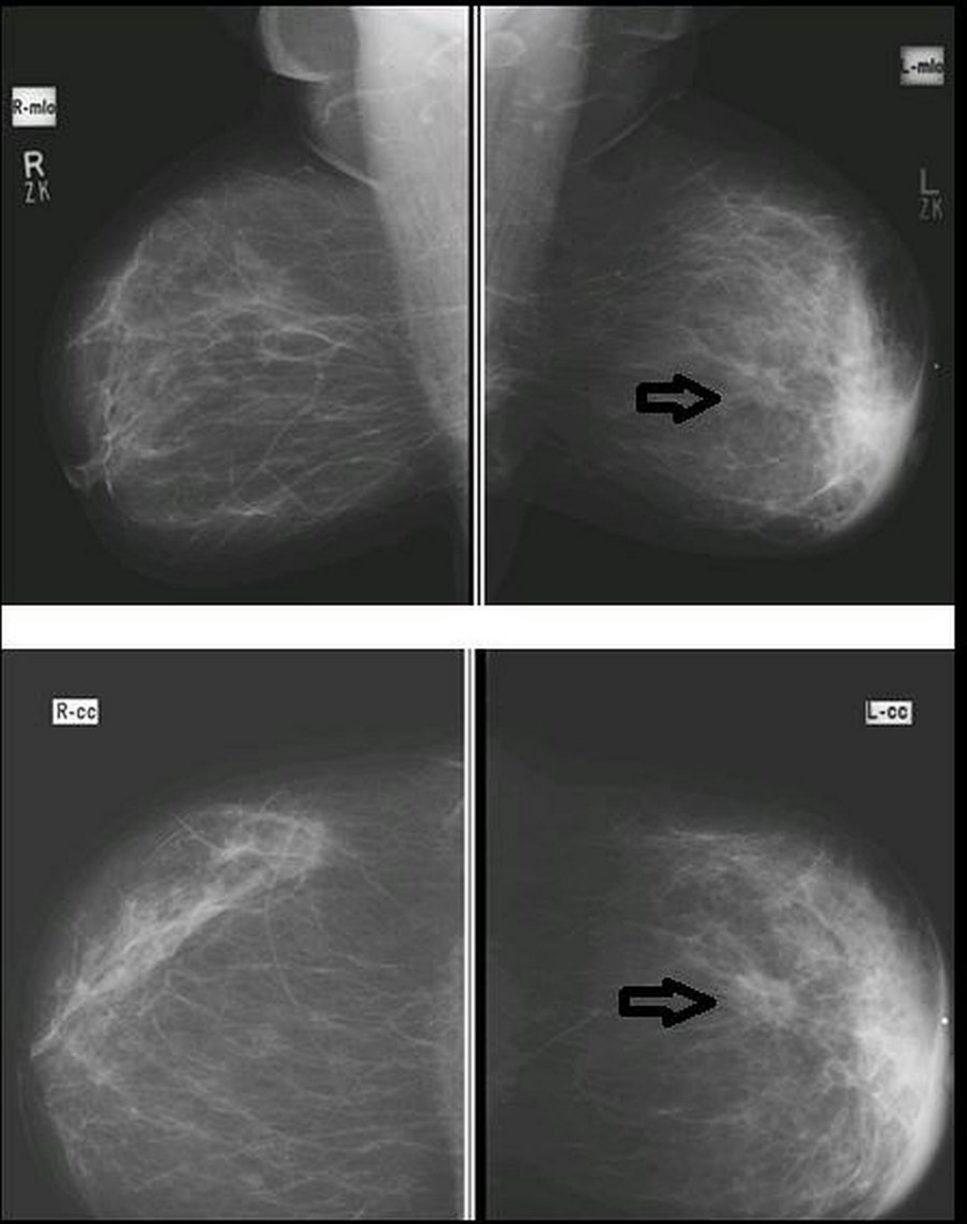
Mediolateral-oblique (MLO) and cranial-caudal (CC) views in a patient with spiculated density (arrow), architectural distortion, skin thickening, and nipple retraction of the left breast, proven to be breast carcinoma on histopathology (true positive).

A total of 122 patients were included in our study according to inclusion and exclusion criteria, with a mean age of 50.79 years (SD +/- 12.06). Among these patients, spiculated density in the mammograms was present in 88 patients (72.1%) (Figure 1).

**Figure 2:**
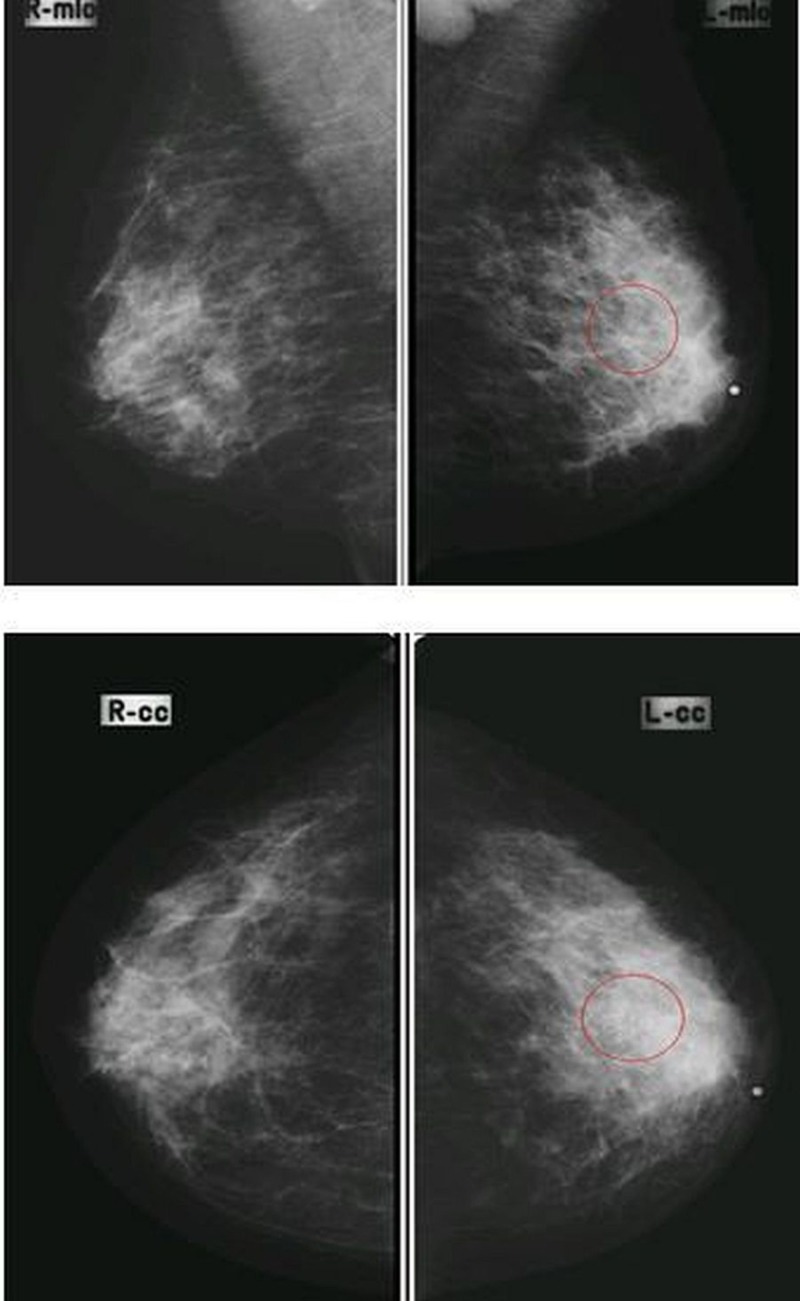
Mediolateral-oblique (MLO) and cranial-caudal (CC) views in a patient having pleomorphic microcalcifications in the upper-outer quadrant of the left breast with enlarged dense axillary lymph nodes, proven to be breast carcinoma on histopathology (true positive).

The sizes of the spiculated density were grouped into either less than two cm or greater than two cm. No spiculated lesion was found in 27.9% of the patients. Lesions that were < 2 cm in size were 18.9% and those > 2 cm were 53.3%. The most common observed site of the lesion was the right upper-outer quadrant 40 (32.8%), followed by the left upper-outer quadrant 34 (27.9%). The pleomorphic microcalcification seen was present in 34 patients (28%) and was absent in 88 (72%). Architectural distortion was seen in 45 patients (36.9%) and was absent in 77 patients (63.1%). Skin features, such as skin thickening and nipple retraction, were present in 22 (18%) and 10 (8.2%) patients, respectively. Axillary lymph nodes were present in 106 patients (86.9%). Similarly, breast cancer in mammograms was present in 100 patients (82%) (Figure 2).

The frequencies of different histopathological features, such as atypical cells, increased mitotic activity, and hyperchromasia were present in 74.6% of the patients and were absent in 25.4%.

A diagnosis of breast cancer on histopathology was seen in 91 patients (74.6%).

Diagnostic accuracy was calculated as follows:

Sensitivity = True Positive (89)/True Positive (89) + False Negative (2) x 100 = 97%

Specificity = True Negative (20)/False Positive (11) + True Negative (20) x 100= 64.5 %

Positive predictive value = True Positive (89)/True Positive (89) + False Positive (11)x100 = 89%

Negative predictive value = True Negative (20) / False Negative (2) + True Negative (20) x 100= 90.9%

Diagnostic accuracy = True Positive (89) + True Negative (20)/True Positive (89) + False Positive (11) + False Negative (2) + True Negative (20) x 100= 89.3% 

A family history of breast cancer was present in 10 patients (8.1%), out of which eight had breast carcinoma (6.5%) and two did not have breast carcinoma (1.6%). The use of hormone replacement therapy (HRT) was present in six patients (4.9%), out of which four had breast carcinoma (3.2%) and two did not have breast carcinoma (1.6%).

## Discussion

Breast cancer has a high prevalence in the community and places very high demands on resources. Historically, ongoing efforts are made to detect it as early as possible by using different clinical and imaging techniques. Self breast examination and routine clinical check-ups are clinical ways of detecting breast cancer early. However, due to the occult nature of most of the early breast cancers, imaging has a vital role to play.

The evolution of mammography dates back to 1913, when surgeon A Salomon conducted a study on the correlation between x-ray findings and histopathology in 3000 mastectomy cases [12]. Conventional mammograms have been replaced by digital mammography, which provides good quality images using reduced radiation doses and can detect breast carcinoma in its earlier stages, resulting in a good prognosis and improved patient survival.

In our study, a total of 122 patients were included according to the inclusion and exclusion criteria. They had a mean age of 50.79 years (SD +/- 12.06) with a minimum age of 31 years and a maximum age of 77 years. Out of the 122 patients, the highest number of patients belonged to the age group between 50 and 60 years (38 patients). The second-highest number of patients belonged to the age group 31-40 years (29 patients). The third-highest group was 40-50 years (25 patients). The patients who had breast cancer on a mammogram, which was later confirmed on histopathology, were of ages ranging from 43-60 years. Iqbal et al., in their study on the survival of patients having locally advanced breast cancer performed in a teaching hospital of Lahore, Pakistan, described the median age of breast cancer patients as 45 years [13]. They also described that most of the women were pre-menopausal, with a receptor-negative disease. Similar statistics were described in other studies performed in developing countries [14].

We calculated the diagnostic accuracy of digital mammography in detecting breast cancer to be 89.3%, with a sensitivity of 97%, a specificity of 64.5%, a positive predictive value of 89%, and a negative predictive value of 90.9%. Pisano et al., in 2008, performed a study and calculated the sensitivity and specificity of digital mammography in different age groups after considering their pre-, peri- or post-menopausal status and breast density [11]. In patients aged < 50 years with a non-dense breast, the sensitivity was 85% with a specificity of 90% while in patients aged > 50 years with non-dense breast, the sensitivity was 66% and specificity was 93% [11]. In our study, the results were similar with a little less specificity, as the number of false positives was high due to a greater degree of suspicion considering the increasing prevalence of breast cancer in our country.

In this study, spiculated density was present in 34 patients (28%) and 27 patients were confirmed to have breast carcinoma on histopathology (27/34, 79.4%). The remaining seven patients having spiculated density on a digital mammogram were proven to have phyllodes tumor (4/7), surgical scarring (2/7), and benign sclerosing lesion (1/7). Most of the patients having spiculated density on digital mammograms had a palpable abnormality, while the rest were detected incidentally. All patients having a palpable abnormality usually had spiculated densities measuring more than two cm (62% cases). Ciato et al. calculated the frequency of spiculated density in his study as 40% (159/397 patients) [15].

The frequency of spiculated density in our study was a little higher due to the late presentation of the patients, as occurs in our country due to less awareness and low socioeconomic status.

The highest number of lesions was located in the right upper outer quadrant (40 cases; 32.8%) with the second-most common location being the left upper-outer quadrant (34 cases; 27.9%), emphasizing the fact of the occurrence of breast cancer commonly in the upper-outer quadrant. This is consistent with the literature results, which also showed that lesions are more common in the upper-outer quadrants. Naeem et al. shared their experience at Lady Reading Hospital, where they included 46 patients having breast cancer; out of them, 26 had lesions in the upper-outer quadrants (26/46, 56.5%) [16].

Pleomorphic microcalcifications were present in 34 cases (28%), out of which 29 (29/34; 85.2%) cases were diagnosed as having breast carcinoma on histopathology. In the past, the literature showed that pleomorphic microcalcification has a positive predictive value of 42% for the detection of malignancy [17]. Our study results are showing a higher positive predictive value of pleomorphic microcalcifications due to the use of digital mammography instead of screen-film mammography, as the former has better contrast resolution and the ability to magnify images digitally.

The indirect features suggesting breast cancer on digital mammograms are architectural distortion, skin thickening, and nipple retraction; these were present in 45 patients (36.9%), 22 (18%), and 10 (8.2%) of patients, respectively.

Overall, our results are in concordance with studies done in the past regarding the frequencies of different mammographic signs in the detection of breast carcinoma. Goedde et al., in his study, found that spiculated density was present in 44% cases, followed by clustered microcalcifications in 25%, mass in 22%, and asymmetric density in 14% of the cases [18].

The presence of axillary lymph nodes on a digital mammogram is not an indicator of breast carcinoma, as it can be seen in normal patients as well as patients having a different diagnosis than breast cancer. Therefore, it is not a criterion for diagnosing breast cancer on mammograms. We calculated the frequency of axillary lymph nodes presence in digital mammograms, which was 87% in 122 patients.

Family history is an important risk factor in the development of breast cancer, especially at an early age. We evaluated the frequency of positive family history in patients having breast cancer, and out of 83 patients, eight had a positive family history (8/83; 9.63%). Similar results have been quoted by past studies, including a study conducted by Chauhan et al., who in a hospital-based descriptive study, calculated the frequency of family history in breast cancer to be 8% [19]. Mai et al. calculated the sensitivity, specificity, and negative and positive predictive values of positive family history for different body cancers in a population-based study. He found that in breast cancer, family history has a sensitivity, specificity, positive predictive value, and negative predictive value of 61.1%. 95%, 61.3%, and 95%, respectively [20].

Hormone replacement therapy (HRT) as a cause of breast cancer is a topic of great debate. It has been postulated that it is associated with a higher incidence of breast cancer in postmenopausal women. This has been based on evidence from three studies, the Collaborative Reanalysis (CR), the Women's Health Initiative (WHI), and the Million Women Study (MWS), which established the casual relationship. We calculated the frequency of HRT use in patients with breast cancer, which was found to be four out of 87 patients (4/87; 4.59%). Czernichow et al. performed a retrospective analysis on the database of Institute Curie where it was found that 1482 patients used HRT for more than six months out of 6737 patients diagnosed with breast cancer (1482/6737; 21.9%) [21]. This difference is because, in our country, hormone replacement therapy is not very popular and not prescribed by physicians on a regular basis.

Considering our study results, we can safely advocate that digital mammography can replace screen-film mammography in our country as well. The most important advantage of using digital mammography is a reduction in the radiation dosage that used to occur with the screen-film technique because of film retakes. Akhtar et al. conducted a study in our department in 2008 comparing the retake rates in conventional versus digital radiography. He concluded that digital radiography had a significantly lower number of retakes (1%) in comparison to the retakes performed in conventional radiography (5.5%) [22].

This study also validates the need for nationwide breast cancer screening programs starting from the age of 40 years and above, as all the patients having breast cancer in our study fall in the range of 44-60 years.

There are a few disadvantages of digital mammography over screen-film mammography. First, the initial cost of installing the system is high as compared to screen-film mammography, but in the long term, it is cost-effective. Second, because digital mammographic equipment has been launched by different manufacturers all around the world, and they are based on different kinds of technologies, it is hard to recommend which one type of digital mammographic equipment should be used. As comparative data is not available yet, it is impossible to recommend, at this point in time, the best digital mammography equipment to be used for the best results [23].

In this study, we also recognized some limitations. First, we excluded those patients having dense breast parenchyma from digital mammograms, as it reduces its sensitivity of detection by digital mammography. But, we kept the heterogeneously dense breast parenchyma, which also reduces the sensitivity of digital mammograms. This might be the fact that may have resulted in false negative cases. Second, we did not stratify the results according to breast density, as many studies in the past have shown different diagnostic accuracies for different degrees of breast density and patterns of parenchyma [11]. Third, we did not compare the diagnostic accuracies of conventional screen-film mammography with those of digital mammography, as has been done in the literature [7]. The fourth limitation is that we did not calculate the interobserver variability, as the digital mammograms were interpreted by a single radiologist.

## Conclusions

To summarize, our data confirmed that digital mammography is a highly accurate tool for breast cancer detection, having a sensitivity of 97%, a specificity of 64.5%, a positive predictive value of 89%, and a negative predictive value of 90.9%, with a diagnostic accuracy of 89.3%. Digital mammography enjoys a few advantages over the film-screen conventional mammography and is replacing the older technique all over the world. However, special attention should be given while selecting equipment, as not much data is available for reference.
